# Differentiation status of primary chronic myeloid leukemia cells affects sensitivity to BCR-ABL1 inhibitors

**DOI:** 10.18632/oncotarget.15146

**Published:** 2017-02-07

**Authors:** Paavo O. Pietarinen, Christopher A. Eide, Pilar Ayuda-Durán, Swapnil Potdar, Heikki Kuusanmäki, Emma I. Andersson, John P. Mpindi, Tea Pemovska, Mika Kontro, Caroline A. Heckman, Olli Kallioniemi, Krister Wennerberg, Henrik Hjorth-Hansen, Brian J. Druker, Jorrit M. Enserink, Jeffrey W. Tyner, Satu Mustjoki, Kimmo Porkka

**Affiliations:** ^1^ Hematology Research Unit Helsinki, University of Helsinki and Department of Hematology, Helsinki University Hospital Comprehensive Cancer Center, Helsinki, Finland; ^2^ Division of Hematology and Medical Oncology, Knight Cancer Institute, Oregon Health and Science University, Portland, OR, USA; ^3^ Howard Hughes Medical Institute, Portland, OR, USA; ^4^ Oslo University Hospital, University of Oslo, Oslo, Norway; ^5^ Institute for Molecular Medicine Finland (FIMM), University of Helsinki, Helsinki, Finland; ^6^ Research Center for Molecular Medicine (CeMM) of the Austrian Academy of Sciences, Vienna, Austria; ^7^ Department of Hematology, St Olavs Hospital, Trondheim, Norway; ^8^ Department of Cancer Research and Molecular Medicine, Norwegian University of Science and Technology (NTNU), Trondheim, Norway; ^9^ Department of Cell, Developmental and Cancer Biology, Oregon Health and Science University, Portland, OR, USA; ^10^ Department of Clinical Chemistry, University of Helsinki, Helsinki, Finland

**Keywords:** chronic myeloid leukemia, high-throughput drug screening, ex vivo, tyrosine kinase inhibitors, CD34

## Abstract

Tyrosine kinase inhibitors (TKI) are the mainstay treatment of BCR-ABL1-positive leukemia and virtually all patients with chronic myeloid leukemia in chronic phase (CP CML) respond to TKI therapy. However, there is limited information on the cellular mechanisms of response and particularly on the effect of cell differentiation state to TKI sensitivity *in vivo* and *ex vivo*/*in vitro*. We used multiple, independent high-throughput drug sensitivity and resistance testing platforms that collectively evaluated 295 oncology compounds to characterize *ex vivo* drug response profiles of primary cells freshly collected from newly-diagnosed patients with BCR-ABL1-positive leukemia (*n* = 40) and healthy controls (*n* = 12). In contrast to the highly TKI-sensitive cells from blast phase CML and Philadelphia chromosome-positive acute lymphoblastic leukemia, primary CP CML cells were insensitive to TKI therapy *ex vivo*. Despite maintaining potent BCR-ABL1 inhibitory activity, *ex vivo* viability of cells was unaffected by TKIs. These findings were validated in two independent patient cohorts and analysis platforms. All CP CML patients under study responded to TKI therapy *in vivo*. When CP CML cells were sorted based on CD34 expression, the CD34-positive progenitor cells showed good sensitivity to TKIs, whereas the more mature CD34-negative cells were markedly less sensitive. Thus in CP CML, TKIs predominantly target the progenitor cell population while the differentiated leukemic cells (mostly cells from granulocytic series) are insensitive to BCR-ABL1 inhibition. These findings have implications for drug discovery in CP CML and indicate a fundamental biological difference between CP CML and advanced forms of BCR-ABL1-positive leukemia.

## INTRODUCTION

All patients with chronic myeloid leukemia in chronic phase (CP CML) and other forms of BCR-ABL1-positive leukemia are currently treated with BCR-ABL1 tyrosine kinase inhibitors (TKIs). CP CML patients experience a relatively normal life span and stay in remission for decades, some even after TKI discontinuation [1, 2]. However, not all patients respond optimally to TKIs and some develop intolerance or secondary resistance. Most cases of TKI resistance are caused by mutations in the kinase domain of the *BCR-ABL1* gene, but some patients develop resistance due to other mechanisms [3, 4]. Although third generation TKIs targeting key gatekeeper mutations have been developed (e.g. ponatinib), there still is a need for novel treatment modalities for suboptimal responders.

*BCR-ABL1*-positive leukemias are classified as CP CML, advanced phase CML, blast phase CML (BC CML) and Philadelphia chromosome-positive acute lymphoblastic leukemia (Ph+ ALL). Several biological and genetic factors are known to differentiate the *BCR-ABL1*-positive leukemias but the basic mechanisms governing the evolution from chronic to blast phase are still mostly unknown [5]. On cell morphology level, CP CML cells consist mostly of differentiated, mature myeloid cells (predominantly neutrophils) with a short half-life, while in BC CML and Ph+ ALL the predominant population consists of undifferentiated, primitive blast cells.

We recently characterized drug responses in BC CML patient samples and cell lines using a high-throughput drug sensitivity and resistance testing platform [6]. In this study we set out to examine drug responses in primary CP CML patient samples collected at the time of diagnosis with the aim of identifying new candidate drugs with efficacy against CP CML cells by BCR-ABL1 dependent and independent mechanisms.

## RESULTS

### Primary CP CML mononuclear cells are insensitive to TKI therapy *ex vivo*

**Figure 1:**
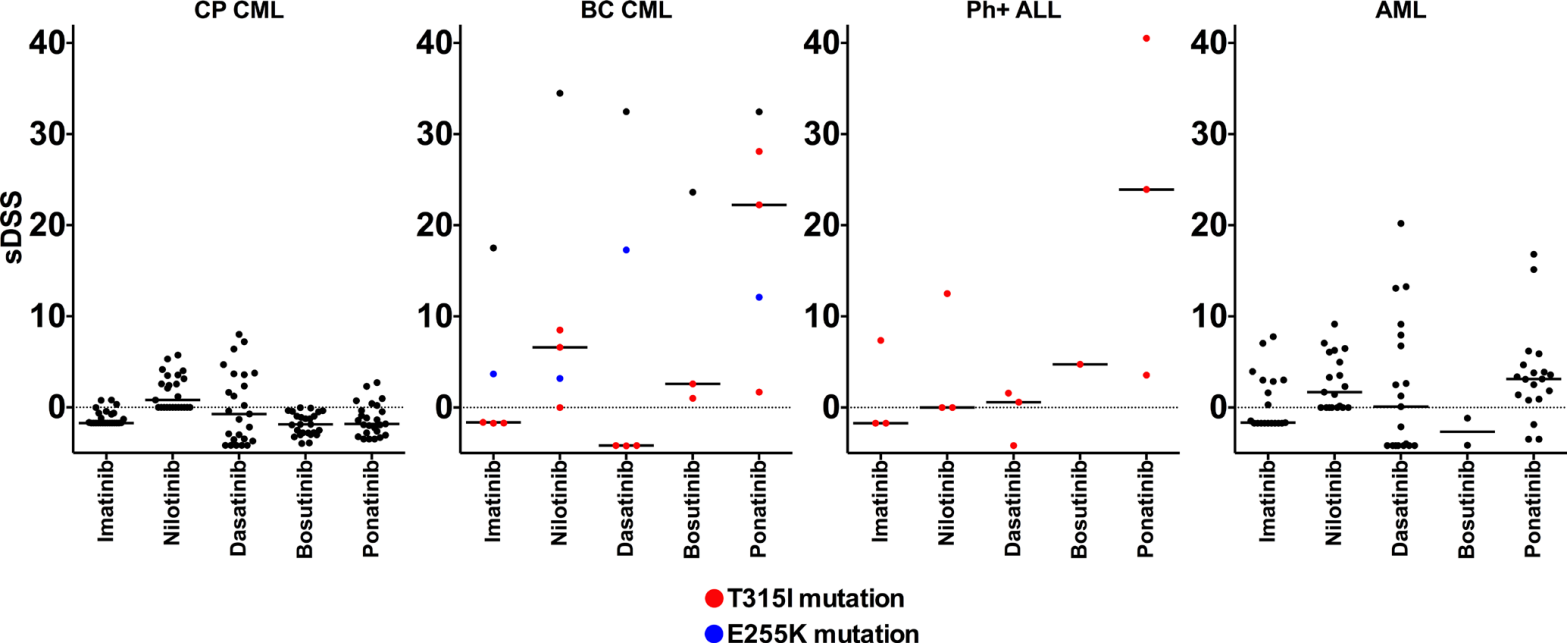
Comparison of *ex vivo* leukemia-specific drug sensitivity scores (sDSS) of common TKIs in CP CML, BC CML, Ph+ ALL and AML samples Colored symbols in BC CML and Ph+ALL graphs denote patients with TKI-resistant T315I (red) or E255K (blue) BCR-ABL1 kinase domain mutation. Lines denote the median sDSS of TKIs.

**Figure 2:**
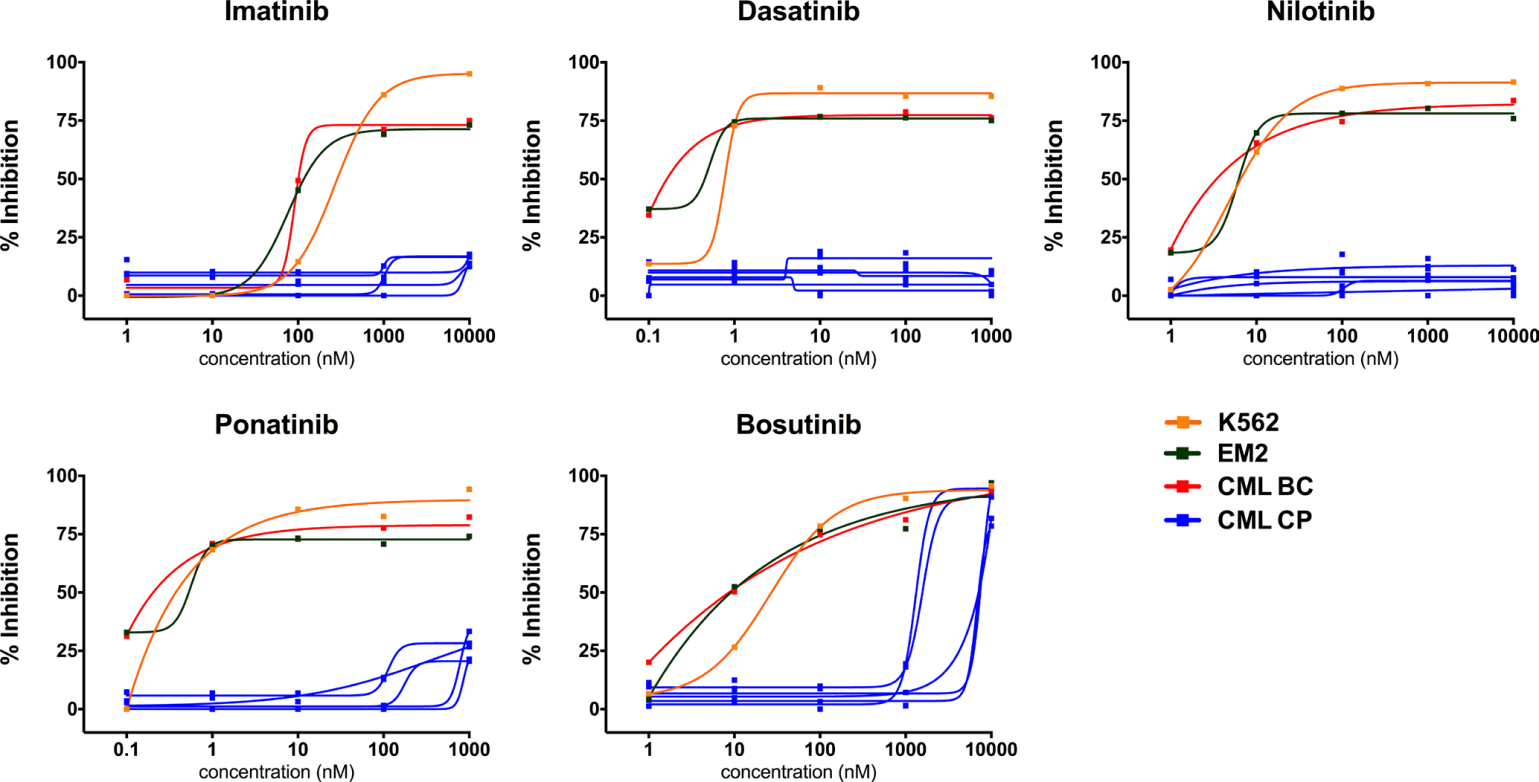
Individual *ex vivo* TKI dose-response curves in different types of CML cell samples Shown are responses from TKI sensitive CML cell lines (K562, EM2), BC CML sample without TKI-resistant BCR-ABL1 mutations and typical CP CML samples from Cohort 1.

We first assessed the *ex vivo* drug sensitivity of fresh primary bone marrow (BM) and peripheral blood (PB) mononuclear cells (MNCs) from 25 CP CML patients using a panel of 295 approved and investigational drugs. The *ex vivo* drug sensitivity was assessed with a sensitivity score taking into account the area under the dose-response curve and normalizing the value to drug responses observed in healthy controls (selective drug sensitivity score, sDSS). Primary CP CML MNCs (*n* = 10 BM and 15 PB) were markedly less sensitive to TKIs and other drug classes *ex vivo* in comparison to BC CML (*n* = 5), Ph+ ALL (*n* = 3), and acute myeloid leukemia (AML, *n* = 20) patient samples (Figure 1). The sDSS values of *ex vivo* responses to the tested common TKIs are depicted in Figure 1. Most Ph+ ALL and BC CML carried the highly TKI-resistant gatekeeper T315I BCR-ABL1 kinase domain mutation, while all CP CML samples were BCR-ABL1 wild type. Representative dose response curves for selected TKIs from individual CP CML, BC CML patient samples and CML cells lines are shown in Figure 2, illustrating almost complete lack of TKI sensitivity *ex vivo* in CP CML samples over a large TKI concentration range. We also tested the *ex vivo* drug sensitivity of CP CML samples using different culture conditions (e.g. different media or incubation times) and sample sources (bone marrow or peripheral blood), with little or no effect on the TKI sensitivities (Supplementary Figure 1). TKI sensitivities were low also when CP CML sample was tested with a cytotoxicity assay (CellTox Green, Promega) in parallel with standard cell viability assay (CellTiter-Glo, Promega) (Supplementary Figure 2). None of the CP CML patients under study showed primary hematological resistance to TKIs *in vivo* and all but one patient (CML CP 23) achieved at least a complete cytogenetic response at 12 months of TKI therapy (Supplementary Table 1). Drug compliance was not formally assessed.

**Figure 3:**
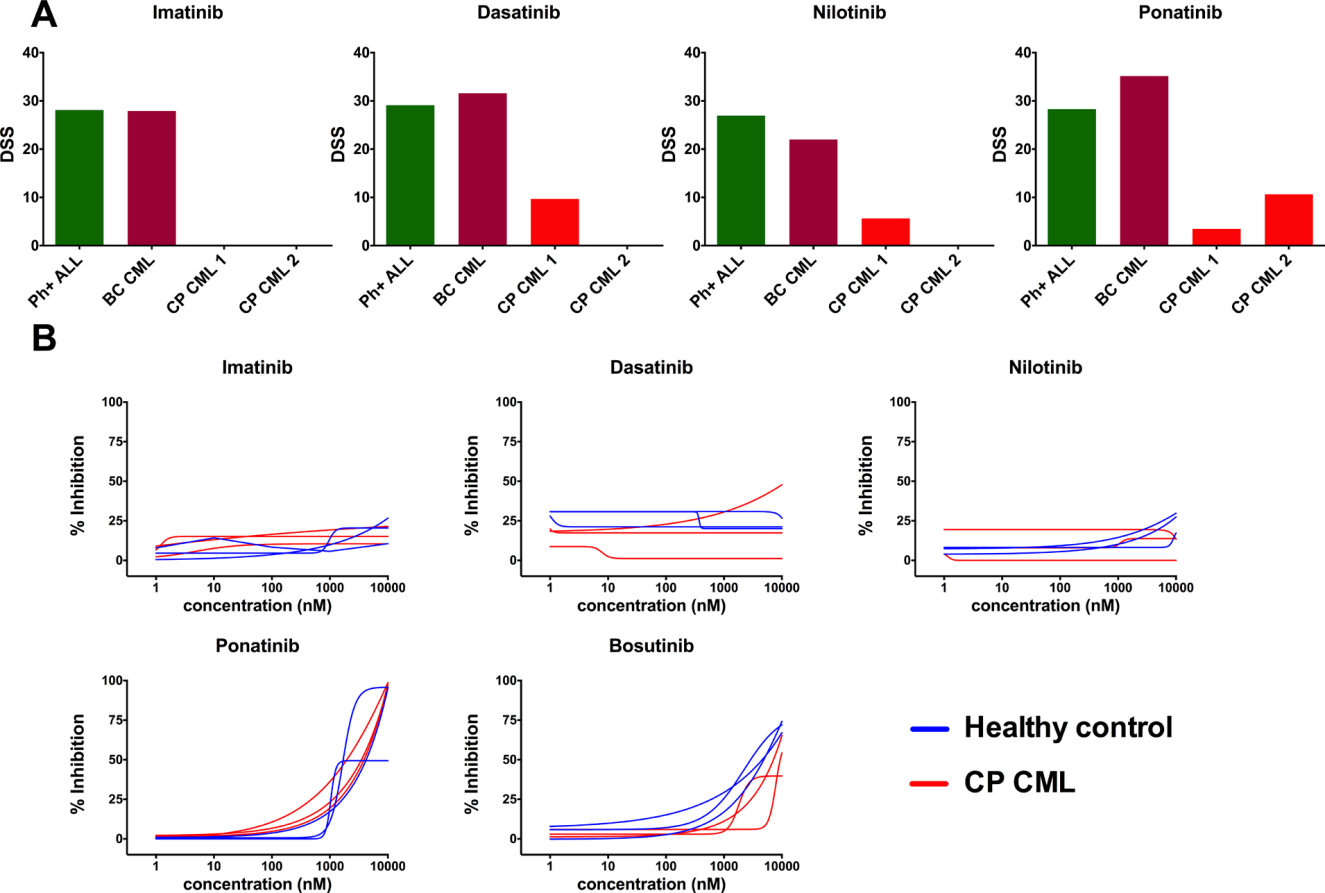
*Ex vivo* TKI sensitivity testing data from Portland (**A**) and Oslo (**B**) validation cohorts and platforms. (A) Drug sensitivity score (DSS) was calculated for Portland samples and DSS of diagnose phase CP CML samples were compared with TKI-sensitive BC CML and Ph+ ALL samples. Tested concentration ranges were 13,72–10000 nM (imatinib and nilotinib) and 1,37–1000 nM (dasatinib and ponatinib). (B) TKI dose-response curves of CP CML (*n* = 3) and healthy BM (*n* = 3) samples were compared in Oslo platform.

Lack of *ex vivo/in vitro* TKI sensitivity was independently confirmed from the Portland and Oslo sample cohorts using distinct but methodologically analogous drug sensitivity platforms. The Portland results showed that both the Ph+ ALL and BC CML samples were sensitive to BCR-ABL1 inhibitors *ex vivo*, whereas CP CML samples showed minimal sensitivity (Figure 3A). Data from the Oslo platform CP CML samples had very similar TKI sensitivity to healthy controls *ex vivo* (Figure 3B).

### Primary CP CML MNCs are relatively insensitive to other oncology drugs *ex vivo*

**Figure 4:**
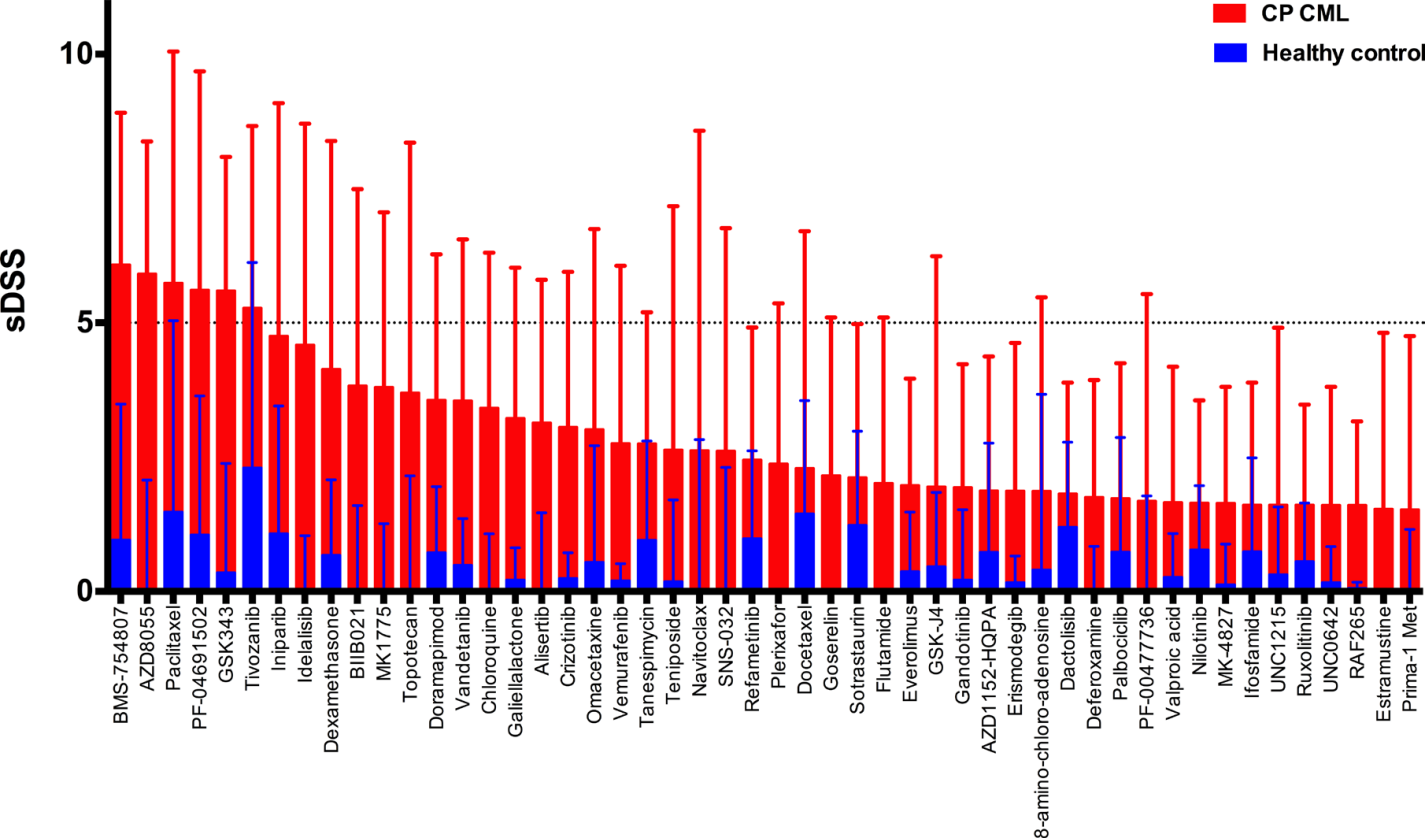
Mean *ex vivo* drug testing results for BM samples from CP CML patients (red bars) and healthy controls (blue bars) Shown are the 50 most leukemia-specific drugs ranked by the selective drug sensitivity score (sDSS). Error bars denote standard deviation.

**Figure 5:**
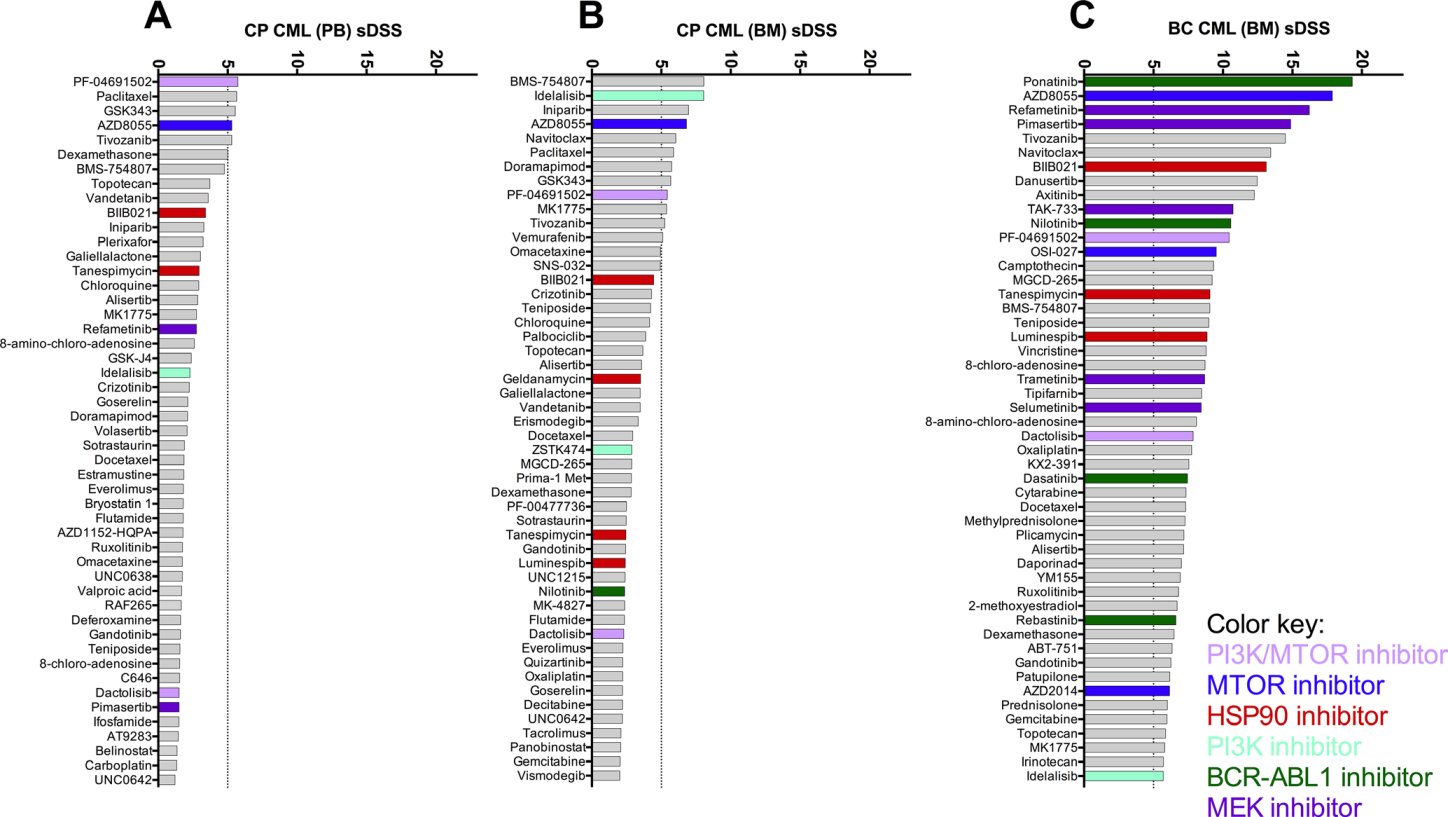
Comparison of overall *ex vivo* drug sensitivity of primary PB CP (**A**), BM CP (**B**) and BM BC CML (**C**) samples. Shown are the 50 most leukemia-specific drugs ranked by the averages of selective drug sensitivity score (sDSS). A selection of effective drug classes is highlighted with colors.

To gain further insight into the drug sensitivity profiles of CP CML MNCs, we assessed the sensitivity of CP CML MNCs to a broad range of drugs commonly used in hematology and oncology (list of drugs provided in Supplementary Table 2). Only 6 out of the 295 (2%) library compounds showed modest activity against CP CML MNCs *ex vivo* (average sDSS score > 5), while most drugs showed sensitivity that was closer to healthy control samples (Figure 4). The IGF-1R inhibitor BMS-754807, mTOR inhibitor AZD8055, mitosis inhibitor paclitaxel, PI3K/mTOR inhibitor PF-04691502, EZH2 inhibitor GSK343 and VEGFR inhibitor tivozanib showed moderate selectivity to CP CML in our drug testing panel (see Supplementary Figures 3 and 4). In contrast, MNCs from BC CML patients showed markedly better overall drug sensitivity compared to CP CML samples in direct comparisons (Figure 5). Clustering of the drug response profiles of patient samples included in this study revealed comparable drug sensitivity profiles between CP CML and healthy control samples with clear segregation from BC CML samples (Supplementary Figure 5). Hence, the observed drug insensitivies of CP CML MNCs *ex vivo* were not restricted to TKIs but were more global.

### TKIs inhibit BCR-ABL1 signaling in primary CP CML MNCs

**Figure 6:**
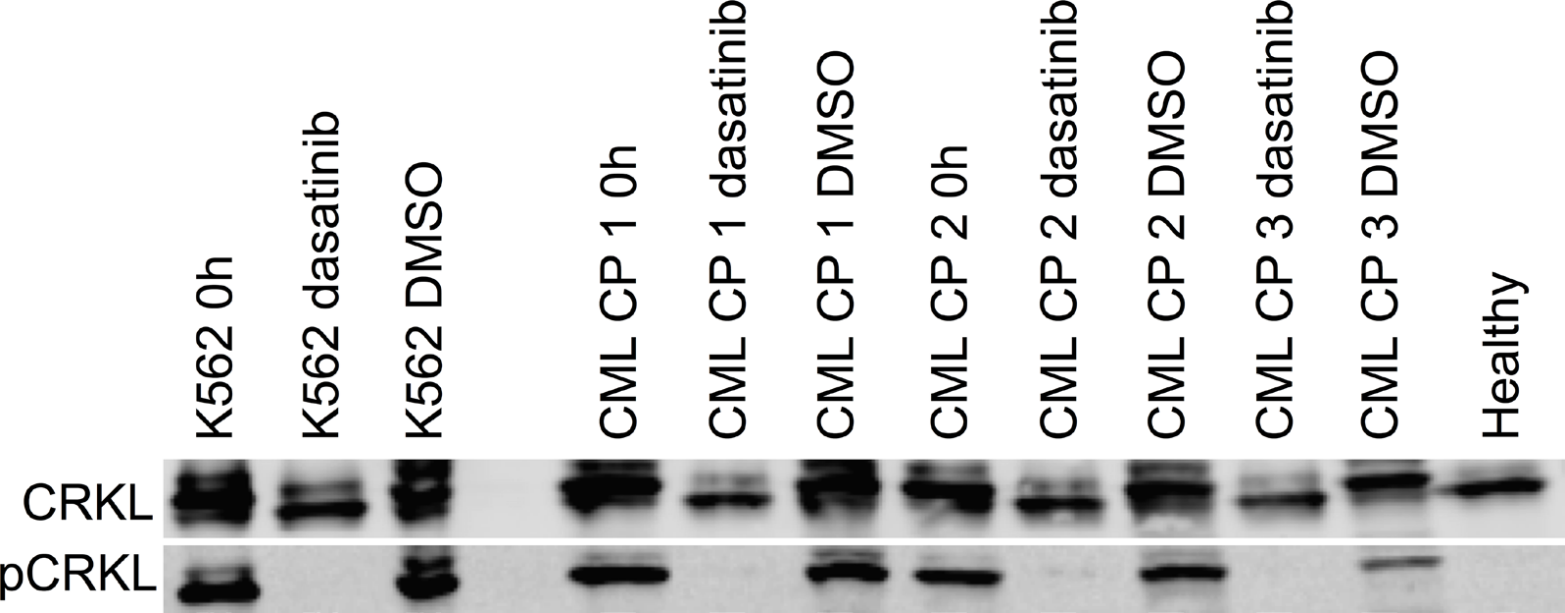
*Ex vivo* effect of dasatinib treatment on primary CP CML MNCs on BCR-ABL1 signaling as measured by phosphorylation of CRKL protein Western blotting was performed on cell lysates before and after 24 h 100 nM dasatinib incubation. 0.1% DMSO was used as a control treatment. CML cell line K562 and healthy BM MNC lysates were used as positive and negative controls, respectively.

We next studied if the consistent lack of responses to TKIs in *ex vivo* drug testing was due to inability of TKIs to block cell signaling downstream of BCR-ABL1 in primary CP CML MNCs. CRKL is a central tyrosine phosphorylated protein detected in cells of patients with CML, suggesting that its association with BCR-ABL1 plays an important role in the pathogenesis of the disease. The specificity of CRKL phosphorylation to BCR-ABL1 signaling, partnered with the stability of the phosphoprotein complex, has established this readout as a robust method to assess BCR-ABL1 status in primary cells and cell lines [7, 8]. We analyzed the effect of dasatinib treatment *ex vivo* on CRKL phosphorylation in three CP CML BM samples of unsorted MNCs using western blotting. Phosphorylation of the CRKL protein was completely inhibited by dasatinib at physiological concentrations in the CP CML MNCs (Figure 6). Thus, despite potent inhibition of BCR-ABL1 signaling in CP CML MNCs by TKIs *ex vivo*, the cellular viability remained unaffected, indicating that alternative pathways govern cellular viability and survival in CP CML MNCs.

### *Ex vivo* TKI insensitivity is linked to cellular differentiation

**Figure 7:**
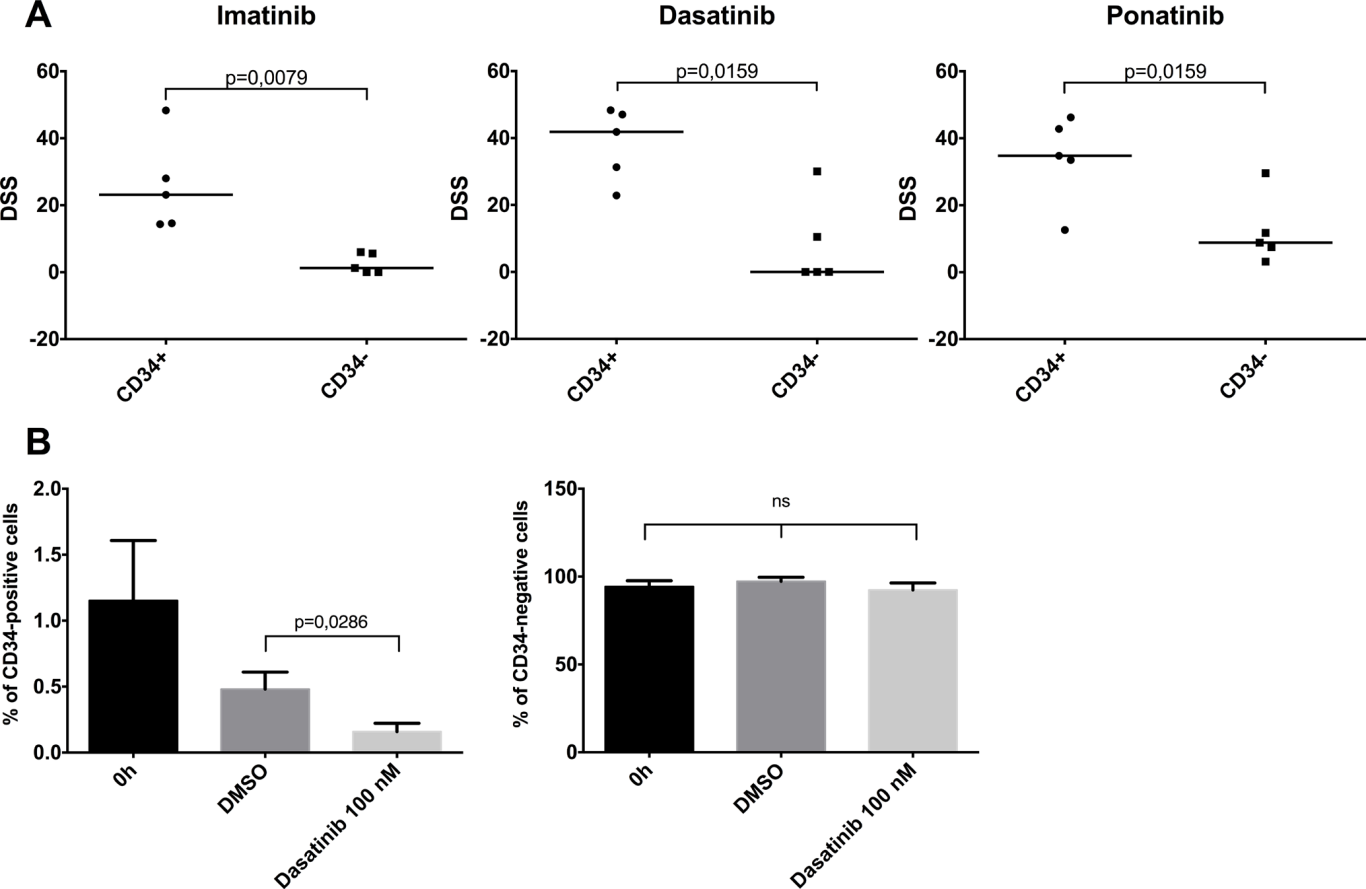
Effect of cellular differentiation on *ex vivo* TKI sensitivity (**A**) Primary BM CP CML MNC samples were sorted to CD34-positive and CD34-negative fractions and were incubated 72 h with imatinib, dasatinib and ponatinib. DSS score (see methods) was used as the viability readout. Lines denote median. (**B**) Unsorted CP CML MNC samples were incubated with 100 nM dasatinib or 0.1% DMSO for 72 h. The CD34-positive and -negative cell percentages were analyzed with flow cytometry. Error bars denote standard deviation. ns, nonsignificant.

In the primary BM and PB samples from CP CML patients used in this study, the vast majority of the BCR-ABL1-positive MNCs were myelocytes, metamyelocytes and neutrophils. To assess whether there is a difference in *ex vivo* drug sensitivity between progenitor and mature cells, we sorted CP CML MNCs into CD34+ and CD34- fractions, and incubated the cells *ex vivo* with imatinib, dasatinib and ponatinib in similar drug testing conditions as performed previously. CD34+ CP CML MNCs were significantly more sensitive to TKIs than CD34- MNCs (Figure 7A), which was most pronounced with the more potent TKIs dasatinib and ponatinib.

We next assessed how dasatinib treatment *ex vivo* affects cell populations in unfractionated MNC samples by flow cytometric analysis. Although 72 h incubation by itself reduced the proportion of CD34+ cells in MNC samples, dasatinib treated wells had significantly lower numbers of CD34+ progenitor cells in comparison to control wells (*p* = 0.03, Figure 7B). CD34- cell proportion remained similar in both groups. Together these data indicate that the dependency of CML progenitor cells upon BCR-ABL1 activity is lost during differentiation into more mature cells.

## DISCUSSION

BCR-ABL1-positive leukemias have been model diseases for the successful development of targeted, safe and efficacious cancer therapies. The advent of TKIs has dramatically changed the outcome of these diseases, in particular for patients with CP CML, many of whom currently have a normal life span [2]. However, cellular mechanisms of response and particularly the effect of cell differentiation state to TKI sensitivity *in vivo* and *ex vivo*/*in vitro* have not previously been explored.

We wanted to utilize the functional *ex vivo* drug testing platforms for discovering new therapies for CP CML patients, similar to our recent studies in BC CML in which we found several promising drug candidates [6]. We prospectively collected a cohort of 25 newly-diagnosed CP CML patients and collected fresh primary PB and BM samples for a comprehensive *ex vivo* drug sensitivity assay platform testing 295 clinical oncology compounds at a broad 4-log concentration range for each sample [9]. Overall, primary MNCs from CP CML patients exhibited reduced sensitivity profiles to the tested drugs compared to our experience with acute leukemias or BC CML samples. Compounds targeting the PI3K/Akt/mTOR pathway had an effect on the viability of CP CML MNCs *ex vivo*, but the leukemia-specificity was modest. The IGF-1R inhibitor BMS-754807, PI3K/mTOR inhibitor PF-04691502 and mTOR inhibitor AZD8055 were the most effective in CP CML patient samples. Increased activity of the PI3K/Akt/mTOR pathway has been proposed as an escape route for TKI resistance in BCR-ABL1-positive leukemias and combining nilotinib with dual PI3K/mTOR inhibitor has been shown to have a synergistic effect [10–12].

The most surprising finding in our study was the significant lack of efficacy of all clinically available ABL TKIs on CP CML MNCs *ex vivo*. We systematically ruled out any technical cause for the insensitivity (e.g. tested different culture media, sample sources, incubation times and conditions, read-outs, concentrations of cells and compounds). Importantly, identical experimental conditions have resulted in good and clinically meaningful drug sensitivities in primary samples from a variety of chronic and acute hematological malignancies we have tested. Samples from patients with BC CML and non-mutated Ph+ ALL were highly sensitive to TKIs *ex vivo*. The results on CP CML MNCs were further validated in two independent patient cohorts and analogous drug testing platforms with the same result: TKIs had minimal effects on the viability of primary CP CML MNCs *ex vivo*. Strikingly, all CP CML patients of the current study cohort responded well to TKI therapy *in vivo*, with all but one patient achieving at least a complete cytogenetic response.

We next tested if TKIs inhibited the BCR-ABL1 signaling pathway in CP CML MNCs *ex vivo*. We showed that dasatinib potently inhibited CRKL phosphorylation in these cells *ex vivo*. Thus, CP CML MNC viability remained unaffected despite efficient inhibition of the canonical BCR-ABL1 signaling by TKIs.

As the MNC samples from diagnostic-phase CP CML patients are composed mostly of myelocytes and other cells from granulocytic series we tested if there was difference in *ex vivo* TKI responses between immature and mature MNCs. By cell sorting experiments, we observed that TKIs were clearly more effective in CD34+ progenitor cell fraction as compared to the more mature CD34- population. Incubation with dasatinib also efficiently decreased the numbers of CD34+ cells, but had little effect on the numbers of cells in the CD34- fraction. Thus in CP CML, the effect of TKIs is derived from targeting the progenitor cell population while the bulk of mature cells with little or no proliferative potential are insensitive to BCR-ABL1 inhibition. However, due to the short half-life of mature myeloid cells, they are eliminated within 2 weeks after the TKI-driven eradication of progenitor cells. This may explain the relatively more rapid initial responses seen in patients with BC CML and Ph+ALL as compared to CP CML patients.

Over the past decade, hundreds of studies have examined the sensitivity of drugs on BCR-ABL1-positive leukemic cells *in vitro* or *ex vivo*. However, the vast majority has been performed on immortalized cell lines, which are derived from and reflect characteristics of advanced BCR-ABL1-positive leukemias (e.g. the BC CML-derived K562 cell line) or on long-term colony assays involving significant modifications for modelling the BM microenvironment [13–15]. In contrast, only a few studies have been performed on primary MNCs collected from CP CML patients. A few studies with imatinib have shown that blocking of BCR-ABL1 signaling induce growth inhibition more effectively in the earlier stages of CML cell maturation [16, 17]. Our study confirms these results and shows that this phenomenon is not limited to only imatinib but applies to other TKIs as well.

Key question for future studies is the isolation of molecular switches governing the differentiation-induced changes in TKI sensitivity from highly TKI-sensitive BCR-ABL1-positive blast-level cells to completely insensitive mature myeloid cells, which comprise the bulk of disease burden in CP CML. If these switches are druggable molecules, we might have a new therapeutic tool for transforming CML blast cells to mature neutrophils which will have a very short life span *in vivo*, similar to differentiation induced by all-trans retinoic acid in acute promyelocytic leukemia.

To conclude, we observed low levels of *ex vivo* drug sensitivity in primary CP CML samples when compared to samples from advanced BCR-ABL1-positive leukemia. Most importantly, CP CML MNC samples showed minimal sensitivity to BCR-ABL1 TKIs *ex vivo* reflecting a differential sensitivity among progenitor and mature CML cells. Our findings have implications for drug discovery in CP CML in achieving treatment-free remission and indicate a fundamental biological difference between CP CML and advanced forms of BCR-ABL1-positive leukemia.

## MATERIALS AND METHODS

### Study cohorts and samples

Patient samples used in this study were derived from 3 independent cohorts. Cohort 1 (Helsinki) was the main study cohort and Cohorts 2 (Portland) and 3 (Oslo) served as validation cohorts.

*Cohort 1 (Helsinki)* consisted of diagnostic phase samples collected from 25 CP CML patients (15 PB, 10 BM samples), 5 patients BC CML (4 BM, 1 PB), 3 patients Ph+ ALL (BM) and from 20 patients with AML (18 BM, 2 PB). BM samples from 9 healthy donors were used as controls. Cohort 1 included diagnostic phase samples from patients participating in the Nordic CML Study Group Clinical first-line study on the combination of pegylated interferon-α2b and dasatinib [18] and were diagnosed, sampled and treated at the corresponding Nordic clinical study centers. The samples were analyzed at the Hematology Research Unit Helsinki and the Institute for Molecular Medicine Finland, University of Helsinki, Finland.

*Cohort 2 (Portland)* consisted of diagnostic phase samples collected from 2 CML patients in CP and from 1 patient in BC, and from 1 patient with Ph+ ALL (all samples from BM). Cohort 2 patients were diagnosed, sampled, and treated and had samples analyzed at the Division of Hematology and Medical Oncology, Knight Cancer Institute, Oregon Health & Science University, Portland, OR, USA. All samples were collected under informed consent using a protocol approved by the Oregon Health & Science University Institutional Review Board.

*Cohort 3 (Oslo)* consisted of BM samples collected from 3 diagnostic phase CML patients and 3 BM samples from healthy controls. Cohort 3 patients were diagnosed, sampled and treated at the Department of Hematology, Oslo University Hospital, Norway, and analyzed at the Department of Microbiology at Oslo University Hospital

All samples were collected after obtaining written informed consent (HRUHLAB2, permit numbers 239/13/03/00/2010, 303/13/03/01/2011, Helsinki University Hospital Ethics Committee). The study was approved by respective local ethical review boards in accordance with the Declaration of Helsinki. MNCs were separated from BM aspirates or PB samples by density gradient centrifugation.

### *Ex vivo* drug sensitivity testing

#### Cohort 1 (Helsinki)

The drug sensitivity and resistance testing (DSRT) protocol used for Cohort 1 samples has been described previously [6, 9]. The drug collection contained 295 different compounds and covered most U.S. Food and Drug Administration/European Medicines Agency (FDA/EMA)-approved anticancer drugs, as well as emerging investigational and preclinical compounds covering a wide range of molecular targets (Supplementary Table 2). The compounds were obtained from the National Cancer Institute Drug Testing Program (NCI DTP) and commercial chemical vendors. Drugs were pre-plated in 384-well plates in 5 different concentrations covering a 10,000-fold concentration range and primary cells added at 10,000 cells per well. All plates were incubated in a humidified environment at 37°C and 5% CO_2_ for 72 h. Cell viability was measured using the CellTiter-Glo luminescent assay (Promega, Madison, WI, US) according to the manufacturer's instructions with a PHERAstar FS (BMG Labtech, Ortenberg, Germany) plate reader. Dose response curves were generated based on the viability readouts and a drug sensitivity score (DSS) was calculated [19]. DSS is an integrative and robust drug response metric based on normalized area under the curve by taking into account all four curve fitting parameters. DSS values were further normalized against the median values from healthy controls (healthy BM MNCs) to obtain a selective DSS (sDSS), which was then used to measure leukemia-specific drug sensitivity for DSRT results. Drugs with sDSS values > 5 were considered effectively selective and > 10 highly selective to tested cells.

#### Cohort 2 (Portland)

The *ex vivo* drug testing platform used for Cohort 2 samples platform was performed using a modification of a protocol described previously [20]. Briefly, 10,000 cells per well were seeded into pre-drugged 384-well plates containing 8-point dose response curves of each drug spanning a ~1000-fold concentration range over 3-fold serial dilutions. Cells were then incubated for 72 h at 37°C and 5% CO_2_. After incubation, cells were subjected to a CellTiter 96 AQueous One solution-based cell proliferation assay (Promega, Madison, WI, USA) and read on a BioTek Synergy 2 plate reader.

#### Cohort 3 (Oslo)

Samples were analyzed as described for Cohort 1, except that the Selleck anticancer library was used for *ex vivo* drug sensitivity screening.

In order to convert the *ex vivo* drug testing data from all 3 platforms used in this study to a common metric, raw sensitivity scores from all samples were analyzed with the same bioinformatics pipeline developed at the Institute for Molecular Medicine Finland [19]. The resulting DSS scores are comparable when analyzing samples within each cohort, but not between cohorts due to small variations in analysis conditions (e.g. viability assays, drug concentration ranges, cell culture media).

### Progenitor cell isolation

To test differences in drug sensitivities between immature and mature CML cell populations, BM MNCs from five Cohort 1 CP CML patient samples were sorted into CD34+ and CD34- fractions using an AutoMACS Separator (Miltenyi Biotec, Bergisch Gladbach, Germany). CD34+ and CD34- cells were seeded in separate groups into predrugged plates containing imatinib, dasatinib and ponatinib. Identical drug sensitivity assay and analysis conditions were used as described above for Cohort 1.

### Flow cytometry analysis

We analyzed the CD34+ cell populations from unsorted BM MNCs of four CP CML patient samples by flow cytometry using a FACSVerse instrument (BD Biosciences, San Jose, CA, USA). MNCs were stained with a FITC labeled CD34 antibodiy (BD Biosciences). A total of 2 × 10^6^ cells per well were seeded in 24-well plates at a density of 1 × 10^6^ cells/ml. Plates were incubated 72 h in the presence of 100 nM dasatinib or 0.1% dimethyl sulfoxide (DMSO) (vol/vol) dissolved in Mononuclear Cell Medium (MCM, PromoCell, Heidelberg, Germany). Sample analysis was done before and after the incubation using the FlowJo software (FlowJo, LCC, Ashland, OR, USA).

### Western blot analysis

Unsorted BM MNCs from 3 CP CML patient samples were seeded in 6-well plates (3 × 10^6^ per well) in MCM and lysed in RIPA buffer before and after 24 h incubation with 100 nM dasatinib or 0.1% DMSO. Protein lysates from the CML cell line K562 and from a healthy volunteer (BM) were used as positive and negative controls, respectively. The protein lysates were loaded to 12% SDS-polyacrylamide gel (Bio-Rad, Hercules, CA, USA). The proteins were transferred to a nitrocellulose membrane (Bio-Rad), after which the membrane was blocked with 5% bovine serum albumin (BSA) for 1 h. Primary phospho-CRKL antibody (Cell Signaling Technologies, MA, USA) was diluted 1:500 to phosphate-buffered saline + 0,05% Tween 20 (PBS-T) + 5% BSA and incubated with the membrane for 1 hour at RT. Secondary infrared 800CW anti-rabbit antibody (LI-COR Biosciences, NE, USA) was diluted 1:15 000 to PBS-T + 5% BSA and incubated with the membrane for 1 h. Total CRKL was measured from the same membrane afterwards with primary CRKL antibody (Santa Cruz, Dallas, TX, USA, 1:1 000) and visualized with the same secondary 800CW antibody. The Odyssey imaging system (LI-COR Biosciences) was used to visualize proteins. 100 nM dasatinib was used for western blot and flow cytometry experiments since this concentration is nearest the average physiological concentration in plasma [21].

### Statistical analysis

The non-parametric Spearman's rank correlation coefficient was calculated with SPSS Statistics software (version 22, IBM, Armonk, NY, USA). All correlation analyses were performed using DSS profiles of the primary patient cell samples. Clustering of the drug sensitivity profiles across patient samples was performed using unsupervised hierarchical complete-linkage clustering using Spearman and Euclidean distance measures of the drug and sample profiles, respectively. Clustering analyses were performed using sDSS profiles of the patient samples from cohort 1. Mann-Whitney *U* test was performed to study the difference between two groups. *P*-values below 0.05 were considered significant.

## SUPPLEMENTARY MATERIALS FIGURES AND TABLES







## References

[1] Huang X, Cortes J, Kantarjian H (2012). Estimations of the increasing prevalence and plateau prevalence of chronic myeloid leukemia in the era of tyrosine kinase inhibitor therapy. Cancer.

[2] Bower H, Björkholm M, Dickman PW, Höglund M, Lambert PC, Andersson TM (2017). Life Expectancy of Patients With Chronic Myeloid Leukemia Approaches the Life Expectancy of the General Population. J Clin Oncol.

[3] Yang K, Fu LW (2015). Mechanisms of resistance to BCR-ABL TKIs and the therapeutic strategies: A review. Crit Rev Oncol Hematol.

[4] Holyoake TL, Helgason GV (2015). Do we need more drugs for chronic myeloid leukemia?. Immunol Rev.

[5] Eide CA, O'Hare T (2015). Chronic myeloid leukemia: advances in understanding disease biology and mechanisms of resistance to tyrosine kinase inhibitors. Curr Hematol Malig Rep.

[6] Pietarinen PO, Pemovska T, Kontro M, Yadav B, Mpindi JP, Andersson EI, Majumder MM, Kuusanmäki H, Koskenvesa P, Kallioniemi O, Wennerberg K, Heckman CA, Mustjoki S (2015). Novel drug candidates for blast phase chronic myeloid leukemia from high-throughput drug sensitivity and resistance testing. Blood Cancer J.

[7] Nichols GL, Raines MA, Vera JC, Lacomis L, Tempst P, Golde DW (1994). Identification of CRKL as the constitutively phosphorylated 39-kD tyrosine phosphoprotein in chronic myelogenous leukemia cells. Blood.

[8] J Hoeve ten, Arlinghaus RB, Guo JQ, Heisterkamp N, Groffen J (1994). Tyrosine phosphorylation of CRKL in Philadelphia+ leukemia. Blood.

[9] Pemovska T, Kontro M, Yadav B, Edgren H, Eldfors S, Szwajda A, Almusa H, Bespalov MM, Ellonen P, Elonen E, Gjertsen BT, Karjalainen R, Kulesskiy E (2013). Individualized Systems Medicine Strategy to Tailor Treatments for Patients with Chemorefractory Acute Myeloid Leukemia. Cancer Discov.

[10] Quentmeier H, Eberth S, Romani J, Zaborski M, Drexler HG (2011). BCR-ABL1-independent PI3Kinase activation causing imatinib-resistance. J Hematol Oncol.

[11] Ding J, Romani J, Zaborski M, MacLeod RA, Nagel S, Drexler HG, Quentmeier H (2013). Inhibition of PI3K/mTOR overcomes nilotinib resistance in BCR-ABL1 positive leukemia cells through translational down-regulation of MDM2. PLoS One.

[12] Okabe S, Tauchi T, Tanaka Y, Kitahara T, Kimura S, Maekawa T, Ohyashiki K (2014). Efficacy of the dual PI3K and mTOR inhibitor NVP-BEZ235 in combination with nilotinib against BCR-ABL-positive leukemia cells involves the ABL kinase domain mutation. Cancer Biol Ther.

[13] Graham SM, Jørgensen HG, Allan E, Pearson C, Alcorn MJ, Richmond L, Holyoake TL (2002). Primitive, quiescent, Philadelphia-positive stem cells from patients with chronic myeloid leukemia are insensitive to STI571 in vitro. Blood.

[14] Corbin AS, Agarwal A, Loriaux M, Cortes J, Deininger MW, Druker BJ (2011). Human chronic myeloid leukemia stem cells are insensitive to imatinib despite inhibition of BCR-ABL activity. J Clin Invest.

[15] Crews LA, Jamieson CH (2013). Selective elimination of leukemia stem cells: hitting a moving target. Cancer Lett.

[16] Oetzel C, Jonuleit T, Götz A, van der Kuip H, Michels H, Duyster J, Hallek M, Aulitzky WE (2000). The tyrosine kinase inhibitor CGP 57148 (ST1 571) induces apoptosis in BCR-ABL-positive cells by down-regulating BCL-X. Clin Cancer Res.

[17] Holtz MS, Slovak ML, Zhang F, Sawyers CL, Forman SJ, Bhatia R (2002). Imatinib mesylate (STI571) inhibits growth of primitive malignant progenitors in chronic myelogenous leukemia through reversal of abnormally increased proliferation. Blood.

[18] Hjorth-Hansen H, Stentoft J, Richter J, Koskenvesa P, Höglund M, Dreimane A, Porkka K, Gedde-Dahl T, Gjertsen BT, Gruber FX, Stenke L, Eriksson KM, Markevärn B (2017). Safety and efficacy of the combination of pegylated interferon-α2b and dasatinib in newly diagnosed chronic-phase chronic myeloid leukemia patients. Leukemia.

[19] Yadav B, Pemovska T, Szwajda A, Kulesskiy E, Kontro M, Karjalainen R, Majumder MM, Malani D, Murumägi A, Knowles J, Porkka K, Heckman C, Kallioniemi O (2014). Quantitative scoring of differential drug sensitivity for individually optimized anticancer therapies. Sci Rep.

[20] Tyner JW, Yang WF, Bankhead A, Fan G, Fletcher LB, Bryant J, Glover JM, Chang BH, Spurgeon SE, Fleming WH, Kovacsovics T, Gotlib JR, Oh ST (2013). Kinase pathway dependence in primary human leukemias determined by rapid inhibitor screening. Cancer Res.

[21] Takahashi S, Miyazaki M, Okamoto I, Ito Y, Ueda K, Seriu T, Nakagawa K, Hatake K (2011). Phase I study of dasatinib (BMS-354825) in Japanese patients with solid tumors. Cancer Sci.

