# Supplementary material for: Differentiation status of primary chronic myeloid leukemia cells affects sensitivity to BCR-ABL1 inhibitors
Source: Oncotarget. 2017 Feb 7;8(14):22606–15. doi: 10.18632/oncotarget.15146 (PMC5410248; doi:10.18632/oncotarget.15146)
Supplement: Supplementary file 1 [file oncotarget-08-22606-s001.pdf]

Differentiation status of primary chronic myeloid leukemia cells affects sensitivity to BCR-ABL1 inhibitors

Supplementary Materials

Supplementary Table 1: Baseline characteristics and response data of Cohort 1 (Helsinki). See Supplementary_Table_1

Supplementary Table 2: DSRT drug library. See Supplementary_Table_2

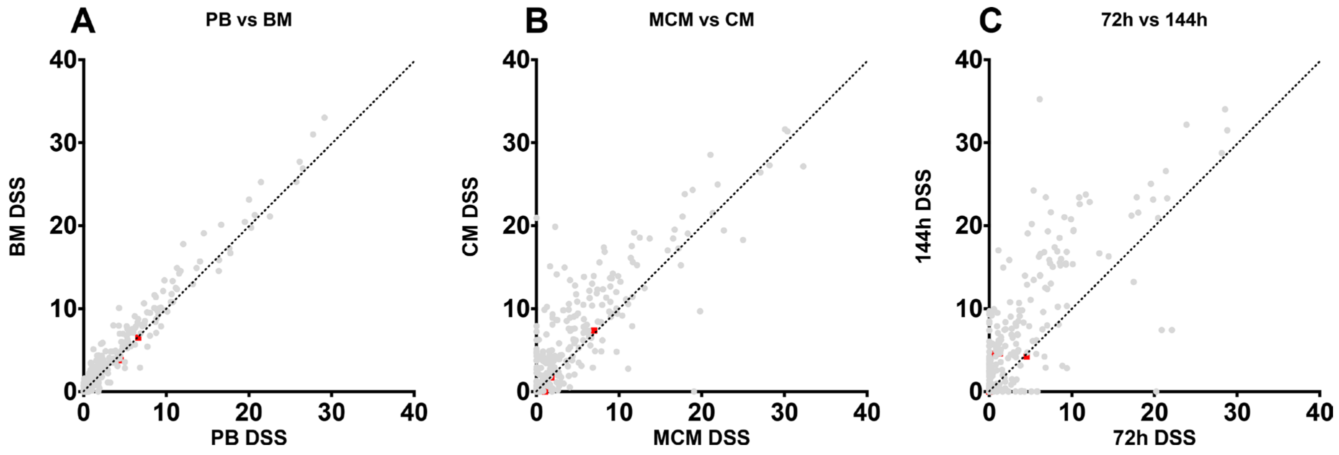

Supplementary Figure 1: Drug sensitivity scores (DSS) of primary CP CML mononuclear cell (MNC) samples from Cohort 1 in different testing conditions. (**A**) Comparison of DSS averages of peripheral blood (PB, *n* = 15) and bone marrow (BM, *n* = 10) samples. (**B**) Comparison of DSS averages of CP CML MNC samples (*n* = 2) tested in different media. (**C**) Comparison of DSS change in one sample tested with 72 h and 144 h incubation. TKI responses are highlighted as red colored squares. MCM, Mononuclear Cell Medium; CM, conditioned medium.

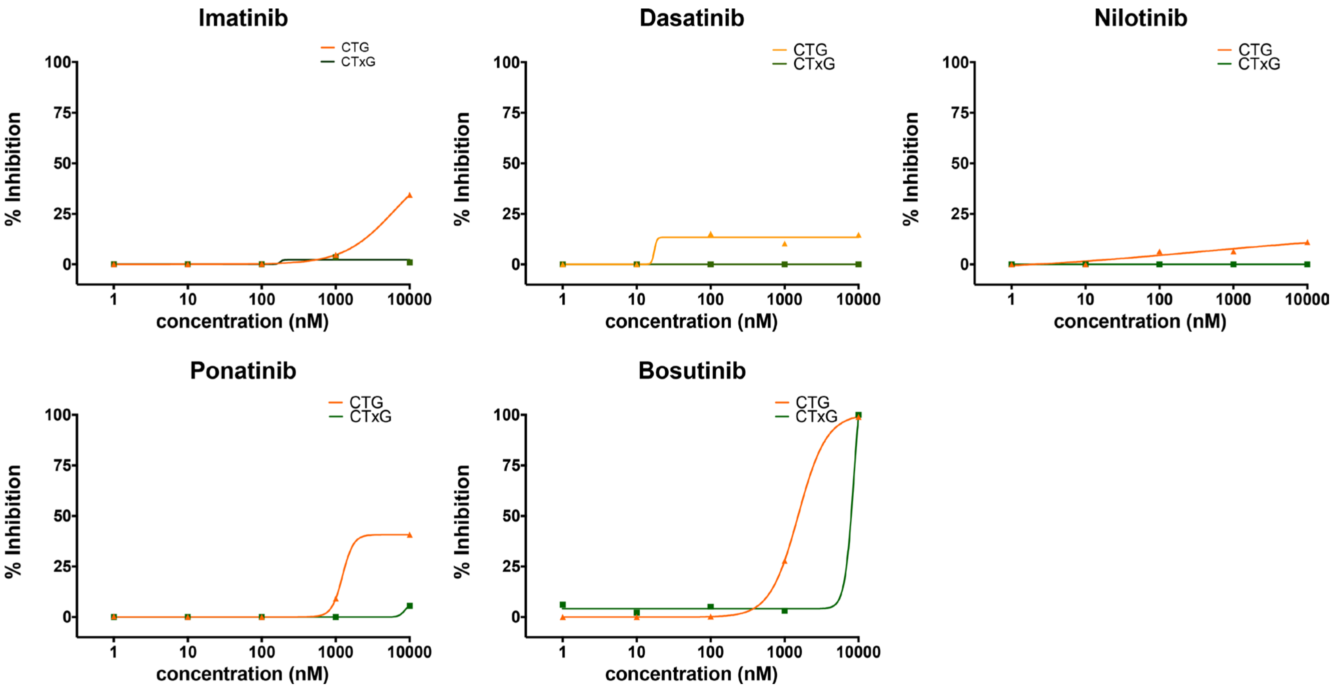

Supplementary Figure 2: Comparison of TKI responses in CP CML MNC with ATP measuring CellTiter-Glo (CTG) and cell cytotoxicity measuring CellTox Green (CTxG) viability assay.

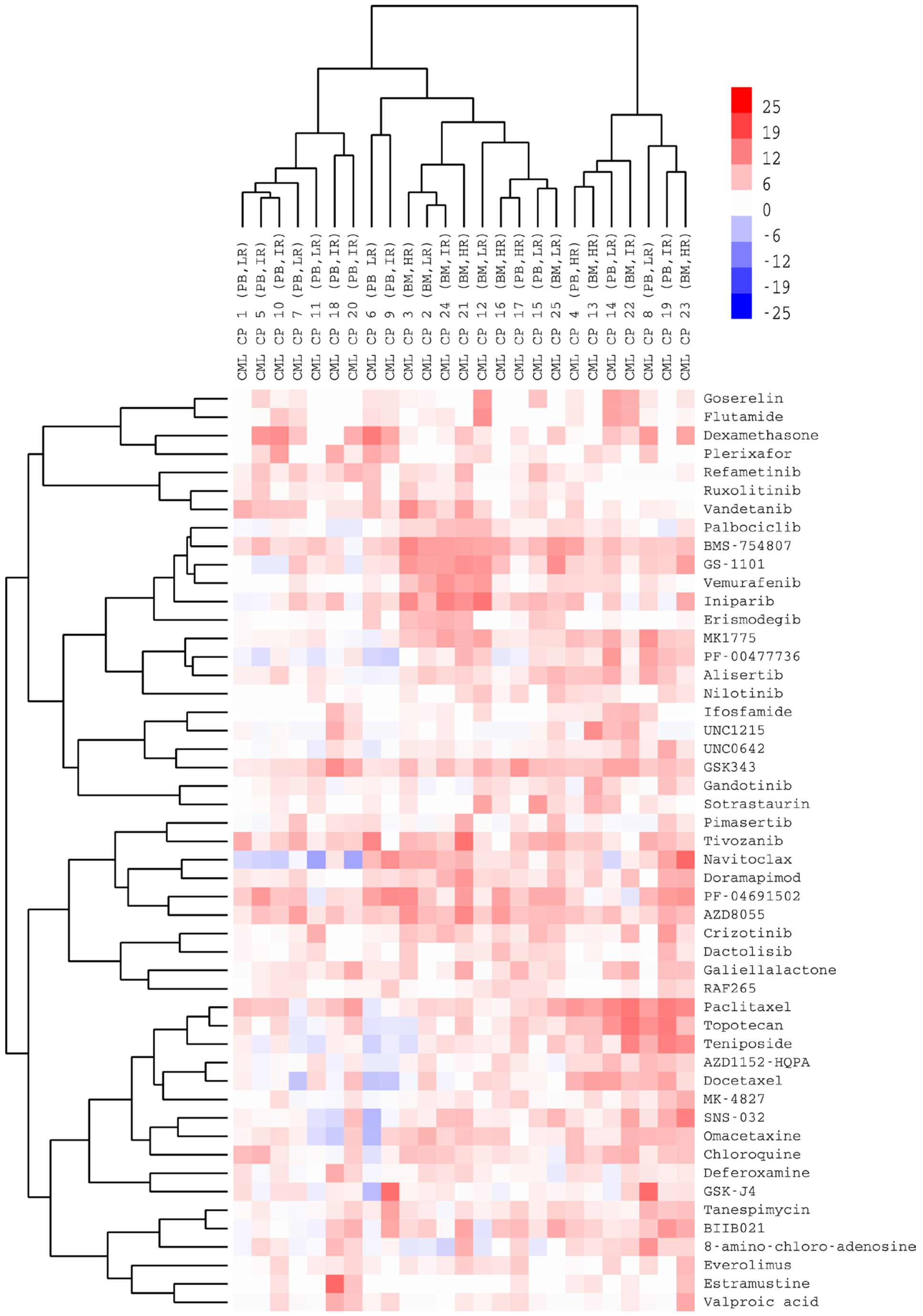

Supplementary Figure 3: Clustering analysis of CP CML samples from Cohort 1 using 50 most sensitive drugs from *ex vivo* drug testing. Color denotes drug sensitivity (red: sensitive, white: neutral, blue: insensitive). Samples were also annotated by Sokal score (low risk: LR, intermediate risk: IR or high risk: HR) and sample source (peripheral blood: PB or bone marrow: BM).

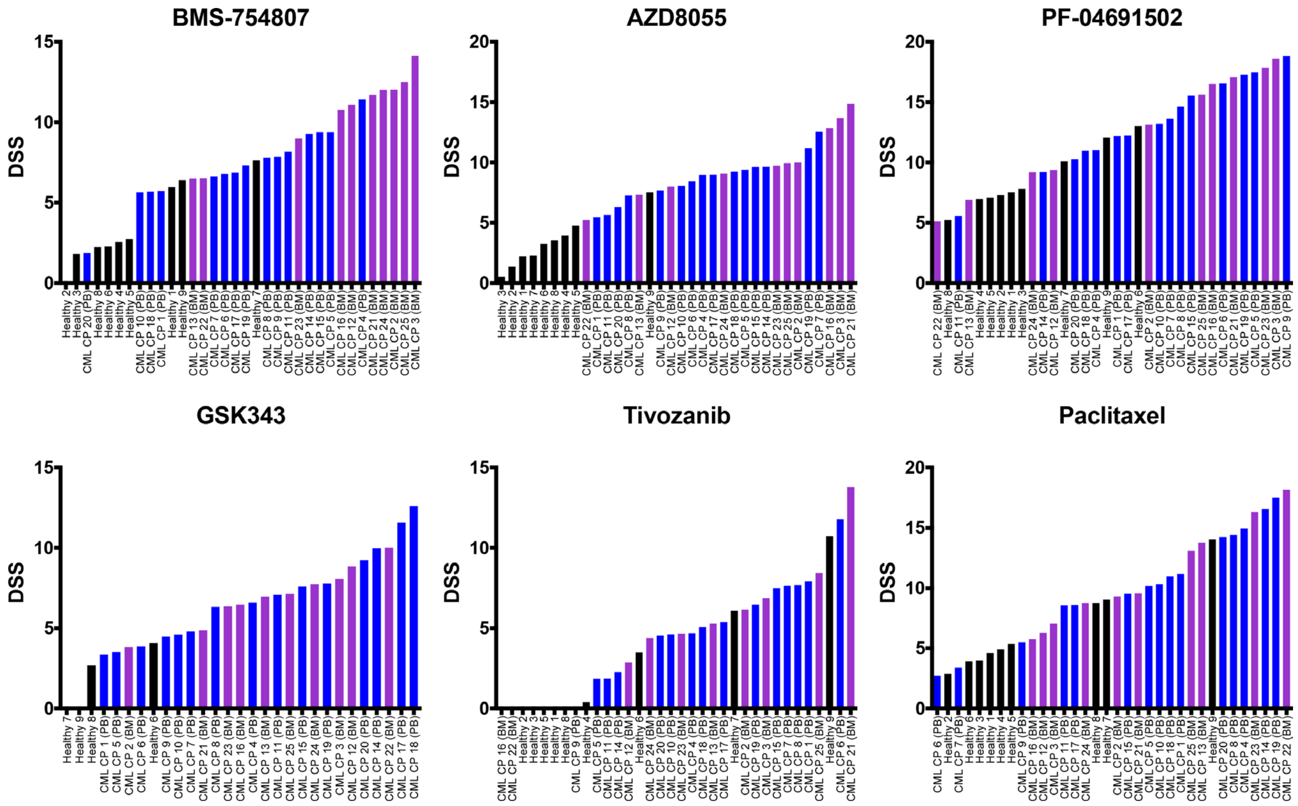

Supplementary Figure 4: Drug sensitivity scores (DSS) of the six sensitive drugs in all CP CML samples studied in Cohort 1. Healthy controls (black), peripheral blood (PB, blue) and bone marrow (BM, purple).

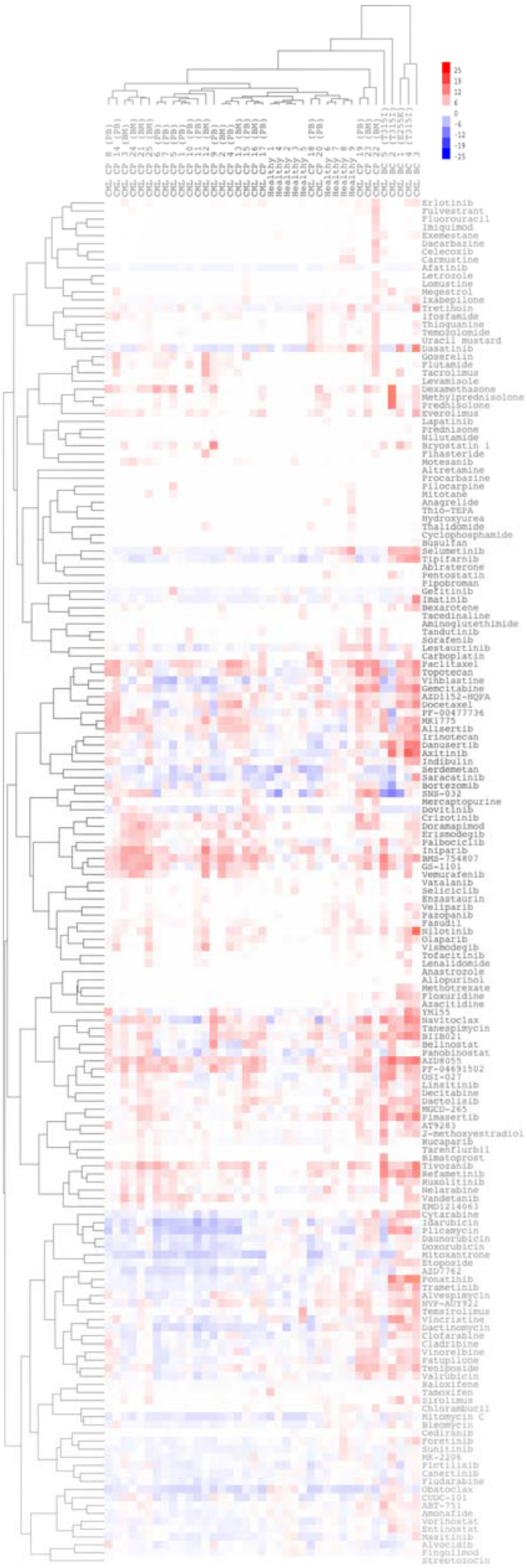

Supplementary Figure 5: Clustering analysis of drug sensitivities in chronic myeloid leukemia chronic phase (CP CML), blast crisis (BC CML) and healthy bone marrow controls. Drugs that were not tested in all samples or showed no activity in any sample were omitted from analysis. Color denotes drug sensitivity (red: sensitive, white: neutral, blue: insensitive). Samples were also annotated by sample type (peripheral blood: PB or bone marrow: BM).
